# Supplementary material for: Bayesian Inference of a Spectral Graph Model for Brain Oscillations
Source: bioRxiv. 2023 Mar 2:2023.03.01.530704. Preprint. [Version 1] doi: 10.1101/2023.03.01.530704 (PMC10002745; doi:10.1101/2023.03.01.530704)
Supplement: Supplementary file 1 [file NIHPP2023.03.01.530704v1-supplement-1.pdf]

bioRxiv preprint doi: https://doi.org/10.1101/2023.03.01.530704; this version posted March 2, 2023. The copyright holder for this preprint (which was not certified by peer review) is the author/funder, who has granted bioRxiv a license to display the preprint in perpetuity. It is made available under aCC-BY 4.0 International license.

Supplementary

Spectral graph model

Notation All the vectors and matrices are written in boldface and the scalars are written in normal font. The frequency f of a signal is specified in Hertz (Hz), and the corresponding angular frequency $\omega = 2\pi f$ is used to obtain the Fourier transforms. The connectivity matrix is defined as $\mathbf{C} = c_{jk}$, where c_{jk} is the connectivity strength between regions j and k, normalized by the row degree.

Mesoscopic model

Given region k out of N regions, we denote the local excitatory signal as $x_e(t)$, local inhibitory signal as $x_i(t)$, and the long-range macroscopic signals as $x_k(t)$. Combining the decay of individual signals, coupling of excitatory and inhibitory signals as well as input white Gaussian noise, the evolution models of $x_e(t)$ and $x_i(t)$ are:

$$\frac{\mathrm{d}x_e(t)}{\mathrm{d}t} = -\frac{f_e(t)}{\tau_e} \star \big(g_{ee}\, x_e(t) - g_{ei}\, f_i(t) \star x_i(t)\big) + p(t)\,, \text{ and,} \tag{3}$$

$$\frac{\mathrm{d}x_i(t)}{\mathrm{d}t} = -\frac{f_i(t)}{\tau_i} \star \big(g_{ii}\, x_i(t) + g_{ei}\, f_e(t) \star x_e(t)\big) + p(t)\,, \tag{4}$$

where $f_e(t)$ and $f_i(t)$ are the ensemble average neural impulse response function, $\star$ stands for convolution, $p(t)$ is input noise, parameters g_{ee}, g_{ii}, g_{ei} are neural gain terms, and parameters τ_e, τ_i are characteristic time constants, which are shared for every region k. We assume Gamma-shaped $f_e(t)$ and $f_i(t)$ as

$$f_e(t) = \frac{1}{\tau_e^2} \exp\left(\frac{-t}{\tau_e}\right) \text{ and } f_i(t) = \frac{1}{\tau_i^2} \exp\left(\frac{-t}{\tau_i}\right).$$

Macroscopic model

Accounting for long-range connections between brain regions, the macroscopic signal x_k is assumed to conform to the following evolution model:

$$\frac{\mathrm{d}x_k(t)}{\mathrm{d}t} = -\frac{1}{\tau_G} f_G(t) \star x_k(t) + \frac{\alpha}{\tau_G} f_G(t) \star \sum_{j=1}^{N} c_{jk} x_j(t - \tau_{jk}^v) + \big(x_e(t) + x_i(t)\big)\,, \tag{5}$$

bioRxiv preprint doi: https://doi.org/10.1101/2023.03.01.530704; this version posted March 2, 2023. The copyright holder for this preprint (which was not certified by peer review) is the author/funder, who has granted bioRxiv a license to display the preprint in perpetuity. It is made available under aCC-BY 4.0 International license.

where, τ_G is the graph characteristic time constant, α is the global coupling constant, c_{jk} are elements of the connectivity matrix, τ^v_{jk} is the delay in signals reaching from the jth to the kth region, v is the cortico-cortical fiber conduction speed with which the signals are transmitted. The delay τ^v_{jk} is calculated as d_{jk}/v, where d_{jk} is the distance between regions j and k and $x_e(t) + x_i(t)$ is the input signal determined from Equations (3) and (4).

SGM only includes 8 global parameters as listed in Table 1. The neural gain g_{ee} is kept as 1 to ensure parameter identifiability. Thus, there are only 7 parameters required to be estimated to determine SGM.

Closed-form model solution in the Fourier domain

A salient feature of SGM is that it provides a closed-form solution of brain oscillations under the frequency domain. Let $\mathcal{F}$ be the Fourier transform at angular frequency $\omega = 2\pi f$. Note that the mesoscopic models for different regions share the same parameters, therefore, without loss of generality, we can drop the subscript k.

The solutions for $x_e(t)$ and $x_i(t)$ under the frequency domain are

$$X_e(\omega) = \mathcal{F}(x_e(t)) = \frac{\left\{1 + \frac{g_{ei}F_e(\omega)F_i(\omega)/\tau_e}{j\omega + g_{ii}F_i(\omega)/\tau_i}\right\} P(\omega)}{j\omega + g_{ee}F_e(\omega)/\tau_e + \frac{(g_{ei}F_e(\omega)F_i(\omega))^2}{\tau_e\tau_i(j\omega + g_{ii}F_i(\omega)/\tau_i)}} = H_e(\omega)P(\omega),$$

and

$$X_i(\omega) = \mathcal{F}(x_i(t)) = \frac{\left\{1 + \frac{g_{ei}F_e(\omega)F_i(\omega)/\tau_i}{j\omega + g_{ee}F_e(\omega)/\tau_e}\right\} P(\omega)}{j\omega + g_{ii}F_i(\omega)/\tau_i + \frac{(g_{ei}F_e(\omega)F_i(\omega))^2}{\tau_e\tau_i(j\omega + g_{ee}F_e(\omega)/\tau_e)}} = H_i(\omega)P(\omega),$$

where $P(\omega)$, $F_e(\omega)$, $F_i(\omega)$ are the Fourier transform of $p(t)$, $f_e(t)$ and $f_i(t)$ at angular frequency ω.

We define the complex Laplacian matrix $\mathcal{L}(\omega) = \mathbf{I} - \alpha\mathbf{C}^*(\omega)$ where $\mathbf{C}^*(\omega) = [c_{ij} \exp(-j\omega\tau^v_{ij})]_{i,j=1,\ldots,N}$. The solution of the macroscopic signals at a angular frequency ω is

$$\mathbf{X}(\omega) = [\,\mathcal{F}(x_1(t)), \cdots, \mathcal{F}(x_N(t))]^{\mathrm{T}} = \left(j\omega + \frac{1}{\tau_{\mathbf{G}}}F_{\mathbf{G}}(\omega)\mathcal{L}(\omega)\right)^{-1} H_{\text{local}}(\omega)\mathbf{P}(\omega), \tag{6}$$

where $H_{\text{local}}(\omega) = H_e(\omega) + H_i(\omega)$.

As SGM provides a closed-form solution $\mathbf{X}(\omega)$, we can compare the modeled and empirical power spectra to estimate the global parameters.
